# Between Blood and Tumor: Retroperitoneal Hematoma Look-Alike Revealed as Diffuse Large B-Cell Lymphoma

**DOI:** 10.7759/cureus.98609

**Published:** 2025-12-06

**Authors:** Usman A Dar, Bobak Zakhireh, Nawar Hakim, Brian Kim, Claudia Didia

**Affiliations:** 1 Department of Radiology, Texas Tech University Health Sciences Center El Paso Paul L. Foster School of Medicine, El Paso, USA; 2 Department of Internal Medicine, Texas Tech University Health Sciences Center El Paso Paul L. Foster School of Medicine, El Paso, USA; 3 Department of Pathology, Texas Tech University Health Sciences Center El Paso Paul L. Foster School of Medicine, El Paso, USA

**Keywords:** diagnostic imaging pitfalls, diffuse large b cell lymphoma, hematoma mimic, misdiagnosed retroperitoneal mass, r chop chemotherapy

## Abstract

Diffuse large B-cell lymphoma (DLBCL) frequently involves the abdomen and retroperitoneum, where it may present as bulky perirenal soft tissue. Spontaneous retroperitoneal hematoma is an increasingly recognized cause of flank pain and anemia, and can produce similar mass-like collections on computed tomography. Distinguishing these entities is crucial, as misclassification can delay potentially curative systemic therapy or expose patients to non-beneficial invasive procedures aimed at hemorrhage control.

We describe a 58-year-old man presenting with progressive left flank and lower quadrant pain, B symptoms, normocytic anemia, acute kidney injury, and mild hypercalcemia. Contrast-enhanced computed tomography demonstrated a large left perirenal retroperitoneal lesion with attenuation in the range expected for acute blood, encasing the kidney and adjacent vasculature and causing mild hydronephrosis. In the context of uncertain minor occupational trauma and mild coagulopathy, a diagnosis of spontaneous retroperitoneal hematoma was favored, and the patient was managed conservatively. Serial hemoglobin measurements showed a progressive decline without hemodynamic instability, prompting catheter-based angiography, which demonstrated no active arterial extravasation or target for embolization. Subsequent renal mass protocol magnetic resonance imaging on hospital day five revealed imaging characteristics more consistent with an infiltrative cellular neoplasm than with evolving hematoma. Image-guided core biopsy established the diagnosis of high-grade B-cell lymphoma with MYC rearrangement, and the patient achieved complete radiologic remission following systemic chemoimmunotherapy.

This case illustrates the substantial clinical and radiologic overlap between retroperitoneal DLBCL and spontaneous hematoma, and emphasizes the limitations of relying on single time point computed tomography in isolation. Extensive rind-like perinephric soft tissue, discordance between anemia and hemodynamic status, absence of an angiographic bleeding source, and the presence of systemic symptoms should prompt reconsideration of a presumed hematoma. A low threshold for advanced cross-sectional imaging and timely tissue diagnosis is essential to avoid diagnostic delay, prevent unnecessary invasive procedures, and expedite appropriate oncologic management when lymphoma underlies an apparent retroperitoneal hemorrhage.

## Introduction

Lymphomas are malignant neoplasms of lymphoid tissue that are broadly categorized into Hodgkin lymphoma and non-Hodgkin lymphoma, with diffuse large B-cell lymphoma (DLBCL) representing the most common aggressive non-Hodgkin subtype in adults and frequently involving abdominal and retroperitoneal sites [1]. On abdominopelvic imaging, DLBCL shows a predilection for mesenteric, para-aortic, pelvic, and perirenal regions, where lymphoma often manifests as bulky nodal or extranodal soft tissue [1,2].

DLBCL arises from mature B cells and typically exhibits rapid growth, which translates clinically into enlarging masses, systemic B symptoms, and laboratory evidence of high tumor burden [1]. Renal and perirenal lymphoma may present as multiple parenchymal nodules, a solitary renal mass, diffuse renal enlargement, or, importantly for the retroperitoneum, a perinephric rind or plaque-like soft tissue that encases the kidney and great vessels while preserving overall organ contours [3,4]. On CT, lymphomatous masses usually demonstrate homogeneous soft-tissue attenuation and mild-to-moderate enhancement; MRI commonly shows T1-hypointense and T2-hyperintense signal with restricted diffusion, reflecting high cellularity and relatively uniform enhancement [1-3].

Accurate staging of DLBCL relies on the integration of cross-sectional imaging, bone marrow evaluation, and functional techniques. Bone marrow biopsy remains an important component of initial staging and risk stratification in selected patients, even in the era of routine FDG PET/CT, which can identify occult marrow involvement that may upstage disease and affect treatment planning [5]. Standard first-line treatment for most patients with DLBCL consists of combination immunochemotherapy with rituximab plus cyclophosphamide, doxorubicin, vincristine, and prednisone, which improves response rates and survival compared with anthracycline-based chemotherapy alone [6]. Histopathologic confirmation with immunohistochemistry is mandatory, and studies with centralized review have shown that expert reassessment frequently leads to reclassification of lymphoma subtype and meaningful changes in management [7].

Within the retroperitoneum, the differential diagnosis of a large soft-tissue mass is broad and includes lymphoma, primary sarcomas, metastatic disease, solitary fibrous tumor, and fibroblastic and myofibroblastic tumors, including inflammatory pseudotumor, as well as non-neoplastic processes such as retroperitoneal fibrosis, hematoma, and inflammatory collections [8,9]. Spontaneous retroperitoneal hematoma is classically associated with anticoagulation, coagulopathy, vascular lesions, or trauma, and typically appears on CT as a hyperattenuating collection that may be lobulated or mass-like and extend along fascial planes [10,11]. Over time, hematomas undergo organization and liquefaction, with a progressive decrease in attenuation and variable internal heterogeneity, which can make them resemble solid neoplasms; conversely, extensive homogeneous lymphoma can mimic a subacute hematoma, particularly when it forms crescentic or mantle-like soft tissue in the perirenal and anterior pararenal spaces [3,4,10,11]. This overlap is compounded when the clinical history is incomplete and laboratory abnormalities are nonspecific.

Distinguishing retroperitoneal lymphoma from retroperitoneal hematoma on CT represents a critical diagnostic challenge with direct implications for management. Misinterpretation of lymphoma as hemorrhage can delay curative systemic therapy and expose patients to unnecessary invasive interventions aimed at presumed bleeding control, such as catheter angiography or empiric embolization, whereas failure to recognize an active hematoma can lead to hemodynamic deterioration [3,4,8-11]. The goal of this case report is to highlight the substantial radiologic overlap between bulky retroperitoneal DLBCL and spontaneous hematoma, illustrate how reliance on a single-timepoint CT examination can be misleading, emphasize CT features that may limit confident differentiation in the absence of clear clinical context, and underscore the need for timely tissue diagnosis to avoid potentially nonbeneficial invasive procedures.

## Case presentation

A 58-year-old man with a remote history of colitis, prior laparoscopic hernia repair, and current dual antiplatelet therapy, but no systemic anticoagulant use, presented to the emergency department with one week of progressively worsening left lower quadrant and left flank pain. The pain radiated to the groin, was exacerbated by movement and deep inspiration, and had become severe enough to limit ambulation and disrupt sleep. He reported subjective fevers, profuse night sweats, nausea with reduced oral intake, and constipation without hematochezia or melena. He denied weight loss, gross hematuria, dysuria, or any prior history of malignancy or bleeding disorders. Although he denied any clearly documented episode of major direct abdominal trauma, he reported a fall at work approximately 1.5 weeks before presentation. He is employed as a manual laborer at a construction site, where his duties frequently involve lifting and transporting heavy objects, as well as forceful pushing and pulling, raising concern for unrecognized minor abdominal trauma during this period. Family history was notable only for a sibling with diabetes. There was no family history of cancer or bleeding diathesis.

On presentation, he was afebrile and hemodynamically stable. Physical examination revealed focal tenderness in the left lower quadrant and left flank without rebound, guarding, or signs of peritonitis. There was no documented costovertebral angle tenderness. Cardiopulmonary and neurologic examinations were unremarkable, and there was no peripheral edema or palpable lymphadenopathy.

Initial laboratory evaluation demonstrated a normocytic anemia, with hemoglobin 11.9 g/dL and hematocrit 35.6%, and biochemical evidence of acute kidney injury, reflected by elevated serum creatinine and blood urea nitrogen. Electrolytes were largely within normal limits aside from mild hypercalcemia. The liver enzymes were normal, with mildly increased total protein and bilirubin. Repeat testing later that morning showed a slight further decline in hemoglobin to 10.2 g/dL and hematocrit to 30.3%, with overall stable renal indices and persistent but mild hypercalcemia. The coagulation studies revealed a mildly prolonged prothrombin time and international normalized ratio with a normal partial thromboplastin time (Table 1).

**Table 1:** Initial laboratory results demonstrating mild normocytic anemia, slightly prolonged PT/INR, azotemia with reduced eGFR consistent with acute kidney injury, hypercalcemia, mild transaminitis, marked LDH elevation, and hypoalbuminemia, which together suggest active systemic disease with renal involvementn WBC = white blood cell count, HGB = hemoglobin, HCT = hematocrit, PLT = platelet count, Na⁺ = sodium, K⁺ = potassium, Cl⁻ = chloride, BUN = blood urea nitrogen, Cr = creatinine, eGFR = estimated glomerular filtration rate, Ca = calcium, AST = aspartate aminotransferase, ALT = alanine aminotransferase, LDH = lactate dehydrogenase, aPTT = activated partial thromboplastin time, PT = prothrombin time, INR = international normalized ratio.

Parameter	Initial Labs	Reference Labs
WBC	4.21 ×10³/µL	4.0 – 11.0 ×10³/µL
HGB	11.9 g/dL	13.5 – 17.5 g/dL
HCT	35.6%	41 – 53%
PLT	361 ×10³/µL	150 – 400 ×10³/µL
Na⁺	139 mmol/L	135 – 145 mmol/L
K⁺	4.2 mmol/L	3.5 – 5.0 mmol/L
Cl⁻	103 mmol/L	98 – 107 mmol/L
Glucose	73 mg/dL	70 – 99 mg/dL
BUN	25 mg/dL	7 – 20 mg/dL
Creatinine (Cr)	2.1 mg/dL	0.6 – 1.3 mg/dL
eGFR	36 mL/min/1.73 m²	≥ 60 mL/min/1.73 m²
Calcium (Ca)	11.0 mg/dL	8.5 – 10.5 mg/dL
AST	50 U/L	10 – 40 U/L
ALT	13 U/L	7 – 56 U/L
Alkaline Phosphatase	67 U/L	38 – 126 U/L
LDH	1,108 IU/L	120 – 246 IU/L
Total Bilirubin	0.5 mg/dL	0.1 – 1.2 mg/dL
Albumin	2.6 g/dL	3.5 – 5.0 g/dL
aPTT	29.8 seconds	25 – 35 seconds
PT/INR	15.7 seconds / 1.2	11 – 13.5 seconds / 0.9 – 1.1

**Figure 1:**
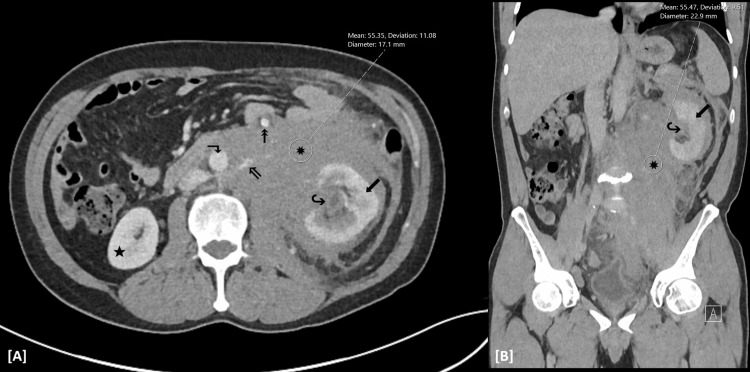
Contrast-enhanced CT of the abdomen in axial (A) and coronal (B) planes at the level of the L3 vertebral body. A large infiltrative soft-tissue density lesion (~55 HU) is present within the left retroperitoneum (✸), forming a rind-like soft-tissue mantle around the left kidney. The lesion encases the left renal vasculature, including the left renal vein (⇖) and renal hilum (↪), with associated left kidney pelvocaliectasis. There is also regional mass effect, with rightward and anterior displacement of the abdominal aorta (↴) and inferior vena cava (IVC) and anterior displacement of the inferior mesenteric vein (↟). Note the diffuse swelling/enlargement of the left renal cortex and delayed nephrogram (⬋) compared with that of the right kidney (★).

**Figure 2:**
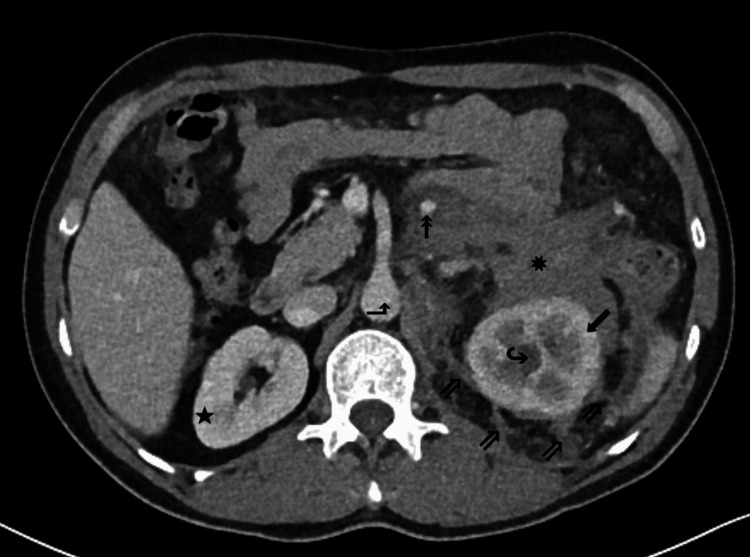
Axial contrast-enhanced abdominal CT at the level of the L1 vertebral body. Left perinephric stranding (⇗) and fascial thickening are present, with a more consolidated appearance of the lesion (✸) anteriorly within the left perirenal space, initially favored to represent a retroperitoneal hematoma. Again noted is delayed enhancement of the left renal cortex (⬋) compared with that of the right kidney (★), with marked left renal pelvocaliectasis (↪), suggestive of a compressive mass effect. At this level, the infiltrative lesion (✸) within the left retroperitoneum encases the inferior mesenteric vein (↟) with relative sparing of the abdominal aorta (⬏).

Contrast-enhanced computed tomography (CT) of the abdomen and pelvis was obtained on the day of admission to further evaluate reported flank pain. Imaging demonstrated a large left-sided retroperitoneal abnormal density centered around the left kidney. The lesion encased retroperitoneal segments of adjacent vascular and ureteral structures, with associated moderate left-sided hydronephrosis and relatively delayed enhancement of the left kidney compared with the right. These findings provided a plausible anatomic correlate for the acute kidney injury observed on presentation. Although the extensive encasement was somewhat atypical for a localized post-traumatic hematoma, in the context of the positive occupational trauma history, use of antiplatelet therapy, and down-trending hemoglobin, a diagnosis of retroperitoneal hematoma was favored initially, although an underlying retroperitoneal malignancy was also considered in the differential diagnosis based on imaging (Figure 1 and Figure 2).

At this point in the patient’s hospital course, general surgery and urology were consulted for the working diagnosis of spontaneous retroperitoneal hematoma. Both services favored observation and serial monitoring of renal function at this time. However, within the next 12 hours, repeat hemoglobin and hematocrit measurements declined to 9.7 g/dL and 28.6%, respectively, demonstrating a downward trend and increasing concern for ongoing bleeding. Over the ensuing days, his hemoglobin and hematocrit fell further to 8.5 g/dL and 26.9%, and then to a nadir of 7.5 g/dL and 22.0%. Notably, despite this progressive anemia, he remained hemodynamically stable without tachycardia or hypotension and did not require any blood transfusion, a discordance that increasingly argued against uncontrolled active hemorrhage.

In light of the progressive decline in hemoglobin, the primary team re-evaluated the likelihood of ongoing hemorrhage and considered additional imaging with either a tagged red blood cell scan or catheter-based angiography. After multidisciplinary discussion with interventional radiology, catheter-based angiography was selected to evaluate for an underlying arterial bleed. However, it demonstrated no active arterial extravasation or pseudoaneurysm, and no embolization was performed (Video 1). The absence of a discrete angiographic bleeding source, when combined with stable vital signs despite continued hemoglobin decline, further reduced the likelihood of a simple, solitary hematoma.

**Video 1:** Digital subtraction aortogram (anteroposterior projection) with a pigtail catheter positioned in the suprarenal aorta, performed to evaluate for the source of a presumed retroperitoneal hematoma. Contrast injection opacifies the major visceral branches, including the celiac trunk, superior mesenteric artery (SMA), and both main renal arteries, with no clear evidence of active extravasation, pseudoaneurysm, or abnormal vascularity to suggest underlying renal mass. In the subsequent parenchymal phase, a delayed and diminished nephrogram is seen on the left compared with the prompt, dense nephrogram on the right, indicating significant hypoperfusion of the left kidney.

**Figure 3:**
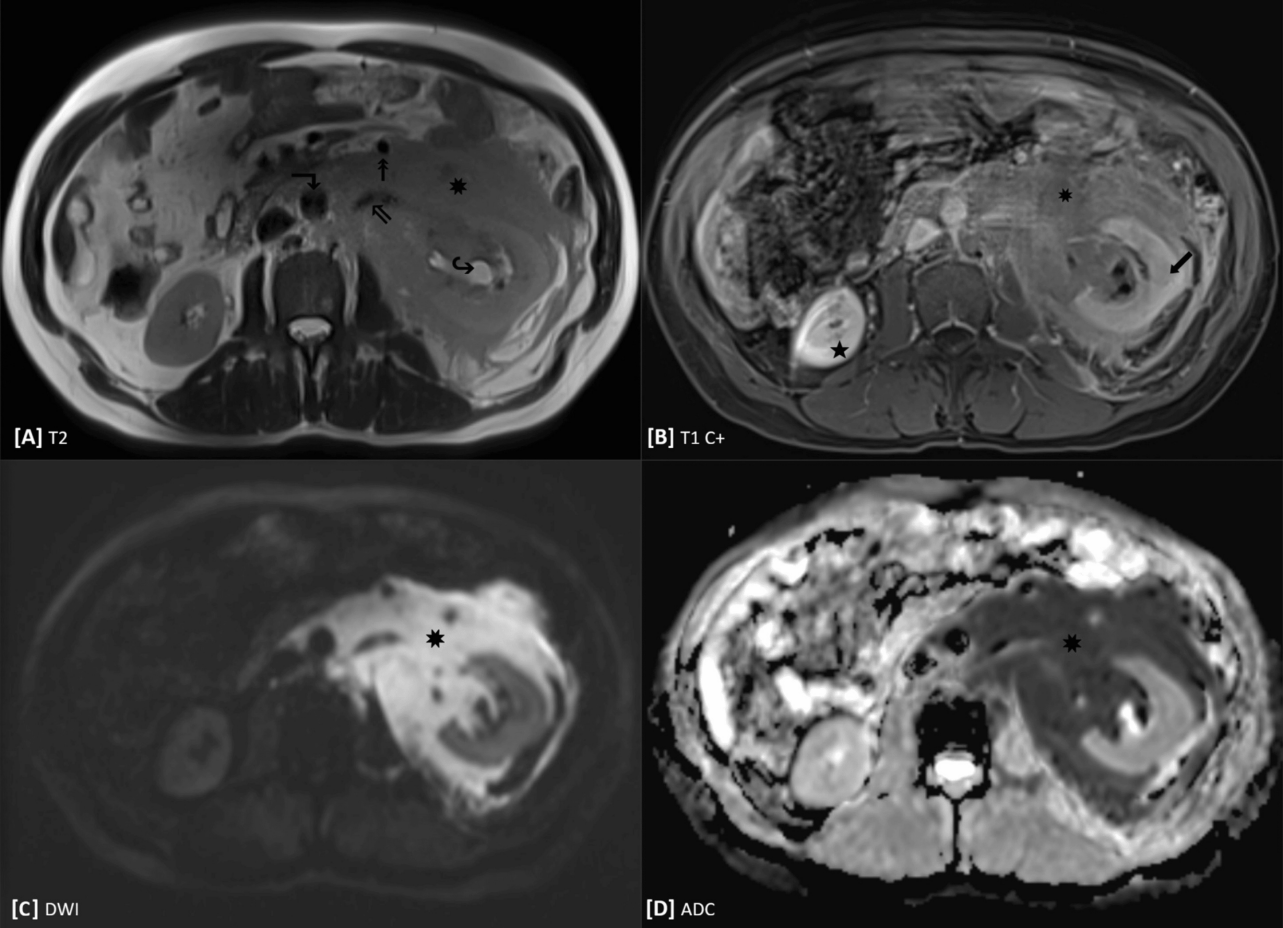
MRI of the abdomen performed with a renal mass protocol. (A) Axial T2-HASTE image redemonstrates a mildly T2-hyperintense left retroperitoneal mass (✸) involving the left perinephric space and extending into the anterior left pararenal space. The mass encases the left renal vein (⇖) with minimal mass effect on the abdominal aorta (↴) and inferior mesenteric vein (↟). Note pelvocaliectasis of the left kidney (↪). (B) Axial post-contrast T1-weighted image demonstrates low-level heterogeneous enhancement of the left retroperitoneal lesion. Again note delayed enhancement of the left renal cortex (⬋) compared with that of the right kidney (★). (C, D) Diffusion-weighted imaging at b = 1000 s/mm² shows high signal intensity within the left perirenal mass, with corresponding low signal on the apparent diffusion coefficient (ADC) map, consistent with marked diffusion restriction and and suggestive of an infiltrative cellular neoplasm.

Following angiography results, a repeat multidisciplinary discussion was held with diagnostic and interventional radiology to further characterize the retroperitoneal process. The team focused on selecting the most appropriate next imaging modality (e.g., MRI versus tagged red blood cell scintigraphy) and on evaluating the need for, and risks associated with, tissue sampling, particularly if the lesion represented predominantly acute or subacute blood. Following this discussion, abdominal MRI was deemed the most appropriate next study. On hospital day five, an abdominal MRI with and without contrast using a renal mass protocol was performed. This study redemonstrated a large infiltrative left retroperitoneal mass-like lesion centered on the left kidney, with imaging features more suggestive of an infiltrative cellular neoplasm than of an evolving hematoma (Figure 3).

**Figure 4:**
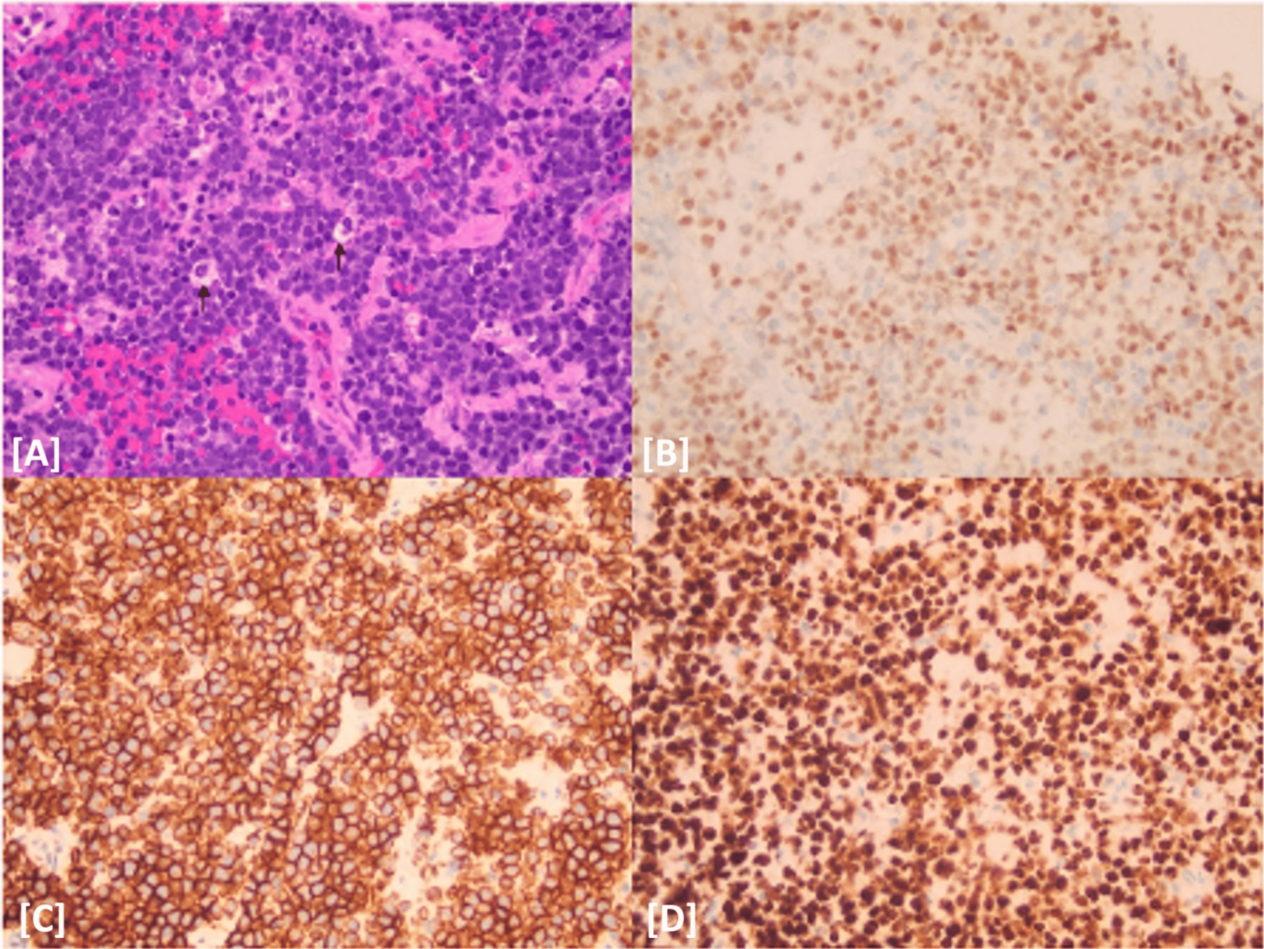
Histopathologic and immunohistochemical findings. (A) H&E stain, original magnification ×400, showing infiltrating malignant lymphoma cells with mild variability in nuclear size and shape and occasional apoptotic bodies (arrows). (B) CD20 immunohistochemical stain, original magnification ×400, demonstrating diffuse staining of the lymphoma cells for the B-cell marker CD20. (C) C-MYC immunohistochemical stain, original magnification ×400, showing nuclear staining of the lymphoma cells for C-MYC. (D) Ki-67 immunohistochemical stain, original magnification ×400, demonstrating a very high proliferative index (approximately 100%) as evidenced by strong nuclear staining for Ki-67, in keeping with a high-grade B-cell lymphoma.. H&E = Hematoxylin and Eosin C-MYC = Cellular myelocytomatosis oncogene

Subsequently, the patient underwent image-guided tissue sampling of the retroperitoneal lesion performed by interventional radiology. Histopathologic analysis revealed a malignant B-cell lymphoma with a MYC gene rearrangement in the absence of BCL2 or BCL6 rearrangements, consistent with a high-grade lymphoma (Figure 4). The patient was started on systemic chemoimmunotherapy with rituximab, cyclophosphamide, doxorubicin, vincristine, and prednisone (R-CHOP). Follow-up imaging obtained seven months after completion of therapy demonstrated complete radiologic remission of the retroperitoneal mass (Video 2).

**Video 2:** Axial contrast-enhanced CT of the abdomen demonstrates a complete radiologic response seven months after completion of R-CHOP therapy for pathologically confirmed diffuse large B-cell lymphoma. This follow-up scan shows no evidence of residual or recurrent disease and resolution of the previously noted lymphadenopathy. CT - Computed Tomography R-CHOP = Rituximab - Cyclophosphamide, Hydroxydaunorubicin, Oncovin, Prednisone

## Discussion

DLBCL is a biologically heterogeneous but potentially curable lymphoma that frequently involves the abdomen and retroperitoneum, whereas spontaneous retroperitoneal hematoma represents a high-risk bleeding complication increasingly encountered in anticoagulated and medically complex patients [1,10,11]. Both entities can present as large retroperitoneal masses on CT, often centered in the perirenal and anterior pararenal spaces, and both may be associated with anemia, flank pain, and nonspecific abdominal symptoms [1,3,4,10,11]. This convergence of clinical and imaging features underlies a central diagnostic problem: a process that appears, on first inspection, to be a hematoma may actually represent extensive lymphoma, and vice versa, with major consequences for therapeutic decision-making. In the present case, initial CT findings and clinical history closely mirrored spontaneous retroperitoneal hematoma, yet very high lactate dehydrogenase (LDH), constitutional B symptoms, progressive anemia in the setting of stable hemodynamics, and subsequent failure to identify an arterial bleeding source emerged as discordant clues that ultimately favored lymphoma over isolated hemorrhage.

From the standpoint of hematoma, spontaneous retroperitoneal hemorrhage has been described in emergency department cohorts, contemporary surgical series, and interventional radiology reports as a complication of anticoagulation, antiplatelet therapy, hypertension, and advanced age, with presentations that include flank pain, abdominal distension, unexplained anemia, and variable hemodynamic instability [10-14]. CT typically shows hyperdense collections in the acute phase, often infiltrating along fascial planes in the perirenal, pararenal, and iliopsoas regions with mass effect on adjacent organs, whereas subsequent imaging may reveal layering, fluid-fluid levels, internal septations, or peripheral enhancement as the hematoma organizes [10-12,14]. Case reports further demonstrate that spontaneous retroperitoneal hematoma can present with sudden hypotension and large-volume bleeding in the absence of trauma or prior interventions, reinforcing the need for rapid recognition and appropriate resuscitation [13].

From a lymphoma perspective, abdominal imaging reviews consistently describe DLBCL as the dominant aggressive non-Hodgkin subtype, with frequent nodal disease along para-aortic and iliac chains and extranodal extension into the kidneys, perinephric space, and adjacent mesentery [1,3]. Renal and perirenal involvement can take the form of multiple nodules, solitary masses, or a circumferential perinephric soft-tissue rind that encases the kidney and proximal ureter, often with preservation of renal contour and only mild mass effect [3,4]. MRI, including contrast-enhanced sequences, supports these CT observations by demonstrating relatively homogeneous low T1 and high T2 signal and uniform enhancement - features that reflect dense, viable tumor and that differ from the time-dependent signal evolution of blood products [2]. Staging series in aggressive lymphomas, including DLBCL and classical Hodgkin lymphoma, emphasize the importance of bone marrow evaluation and whole-body functional imaging for accurate assessment of disease extent and risk stratification [5,15,16]. PET/CT-based assessment of nodal status improves detection of supra- and infra-diaphragmatic disease, including pelvic and retroperitoneal pathways, and can reveal metabolically active retroperitoneal masses that might otherwise be attributed to non-neoplastic causes on morphologic criteria alone [15,16].

At the same time, several imaging-based analyses highlight that lymphomatous sheets in the retroperitoneum may mimic benign fibroinflammatory processes. Algorithms for retroperitoneal masses describe plaque-like, ill-defined soft tissue encasing the aorta, iliac vessels, and ureters in both lymphoma and primary retroperitoneal fibrosis, with only subtle differences in enhancement, symmetry, and caudal extension [8,9]. Additional series exploring retroperitoneal fibrosis and its malignant mimics reinforce that mantle-like soft tissue around the great vessels may represent a spectrum that includes idiopathic fibrosis, Erdheim-Chester disease, or infiltrative malignancy, with overlapping CT and MRI appearances and variable FDG avidity on PET/CT [17,18]. Multidetector CT studies further demonstrate that location, craniocaudal extent, and the pattern of vascular encasement can help differentiate lymphoma presenting as a retroperitoneal mass from retroperitoneal fibrosis, but still leave considerable room for diagnostic ambiguity [19]. In this setting, reliance on attenuation and pattern alone becomes hazardous.

Spontaneous retroperitoneal hematoma introduces a second layer of complexity to this overlap. Large, heterogeneous or partially organizing hematomas may appear mass-like on CT, and several series and case reports describe situations in which hyperattenuating retroperitoneal collections were initially interpreted as a neoplasm, as well as renal or perirenal lymphoma that was misdiagnosed as isolated hematoma [3,4,10-14]. These observations show that hemorrhage and tumor can coexist or evolve in sequence within the same anatomic compartment, and that a single cross-sectional snapshot may not reliably distinguish between them.

Comparing the two entities throughout the diagnostic workup highlights both similarities and discriminators. In DLBCL, laboratory evaluation often reveals elevated lactate dehydrogenase, high inflammatory markers, and sometimes cytopenias or hypercalcemia, whereas coagulation parameters may be normal unless there is liver dysfunction or prior treatment [1]. In spontaneous retroperitoneal hematoma, the dominant abnormalities are anemia and coagulopathy or therapeutic anticoagulation, although inflammatory markers and LDH may be nonspecifically elevated in both conditions [10-12,14]. On ultrasound, lymphoma tends to manifest as hypoechoic solid masses or nodal conglomerates, whereas hematomas are often complex, heterogeneous, and evolving; however, sonographic windows in the retroperitoneum are frequently limited [1,10,11]. CT remains the primary modality for both. Lymphomatous masses are usually homogeneous, iso- to slightly hypoattenuating relative to muscle, with mild enhancement, vessel encasement, and preservation of fat planes in early disease, whereas acute hematomas are hyperattenuating and nonenhancing, with an evolution toward lower attenuation and possible peripheral or septal enhancement over time [1,3,4,8,10-12,14]. MRI can aid differentiation by exploiting differences in the temporal evolution of blood products compared with the relatively stable T1- and T2-signal characteristics of lymphoma, although chronic hematomas and fibrotic masses may still resemble solid tumors [2,8,9,17-19]. PET/CT generally shows intense FDG uptake in viable lymphoma, whereas non-neoplastic processes such as mature fibrosis and uncomplicated hematoma more often demonstrate lower or absent uptake, although inflammation and organizing blood can produce intermediate activity [15-18].

Histopathologic analysis remains the ultimate arbiter. In DLBCL, tissue sampling must provide sufficient material for morphology, immunohistochemistry, and, ideally, molecular analysis. Expert review has been shown to reassign lymphoma subtype and alter treatment intent in a substantial fraction of cases, underscoring the risk of relying on limited or poorly targeted samples [7]. In our case, image-guided core biopsy demonstrated a CD20-positive, MYC-rearranged B-cell lymphoma with a nearly 100% Ki-67 proliferation index and no BCL2 or BCL6 rearrangements.

For deep retroperitoneal lesions, percutaneous image-guided core biopsy is standard when safe, and contrast-enhanced CT-guided core techniques have demonstrated a high diagnostic yield for malignant retroperitoneal masses, including lymphoma [20]. Endoscopic ultrasound-guided fine-needle aspiration or fine-needle biopsy provides an alternative route for lesions abutting the gastrointestinal tract and can achieve a high diagnostic yield for lymphoid and non-lymphoid malignancies [21-23]. These percutaneous and endoscopic techniques are particularly useful when CT suggests a retroperitoneal process that might otherwise require laparotomy for diagnosis. In contrast, suspected spontaneous retroperitoneal hematoma is usually managed without biopsy; concerns about provoking additional bleeding often preclude tissue sampling unless there is strong evidence of an underlying mass [10-14].

Management strategies further illustrate the divergence between DLBCL and spontaneous retroperitoneal hematoma. For DLBCL, multi-agent immunochemotherapy with rituximab plus CHOP remains the backbone of first-line therapy and can lead to long-term remission or cure in a large proportion of patients [6]. Treatment intensity and field selection are guided by stage, performance status, and extranodal involvement, which depend critically on accurate initial imaging and marrow assessment [5,15]. In contrast, contemporary management of spontaneous retroperitoneal hematoma emphasizes hemodynamic stabilization, correction of coagulopathy, and careful selection between conservative treatment and early transarterial embolization [10-12,14]. Observational cohorts and interventional series show that many hemodynamically stable patients without active contrast material extravasation can be managed nonoperatively, whereas those with ongoing transfusion requirements, imaging evidence of active bleeding, or hemodynamic compromise benefit from prompt catheter-based embolization, often targeting multiple lumbar and iliolumbar branches [11,12,14]. Surgical exploration is now reserved for refractory or compartment-like presentations or when associated pathology such as a ruptured tumor or aneurysm is suspected [10-13]. Advanced adjuncts such as intravascular ultrasound can clarify the relationship between retroperitoneal malignancies and major vessels, assisting in operative planning and avoiding unnecessary vascular sacrifice [24].

Taken together, the literature indicates that DLBCL and spontaneous retroperitoneal hematoma share a predilection for the retroperitoneal space and can both present as large, homogeneous or mildly heterogeneous perirenal and pararenal collections on CT that narrow adjacent vessels and displace bowel [1,3,4,8-12,14,19,24]. Overlapping attenuation, enhancement, and growth patterns - particularly when imaging is obtained at a single time point and clinical information is incomplete - can obscure the distinction between hemorrhage and malignancy. At the same time, differences in FDG avidity in PET/CT, MRI signal evolution, and the presence of ancillary findings such as additional nodal disease, organ infiltration, or clear temporal change remain important clues [1-3,8,9,15-19]. The combined evidence supports a low threshold for early tissue diagnosis when a presumed retroperitoneal hematoma exhibits atypical morphology, extensive perinephric rind-like soft tissue, or discordant clinical features, since misclassification may either delay curative systemic therapy for DLBCL or expose patients with true hemorrhage to inappropriate procedures and treatment algorithms designed for neoplastic disease [3,4,7,10-12,20-23].

## Conclusions

This case underscores that retroperitoneal diffuse large B-cell lymphoma can present as a seemingly homogeneous perirenal collection mimicking spontaneous hematoma, yet requires a fundamentally different treatment pathway. Diagnosis relies on recognizing key red flags: a persistent rind-like soft-tissue component, a mismatch between anemia severity and hemodynamic stability, and the absence of an active bleeding source. When a presumed hematoma displays these atypical features, early tissue confirmation should be prioritized over repeated hemorrhage-centered investigations. Integrating these clinical and radiologic discrepancies into the initial assessment allows for timely diagnosis and creates a bridge to appropriate systemic therapy, sparing patients unnecessary invasive procedures.
